# Frailty Is a Predictor of Inpatient Mortality and Unplanned ICU Admission Following Early Fixation of Intertrochanteric Fractures

**DOI:** 10.1007/s43465-026-01751-z

**Published:** 2026-03-12

**Authors:** Victor Koltenyuk, Jared Sasaki, Nithin Gupta, Zachary Chanmin, Ruby G. Patel, Taylor Manes, Jack W. Weick

**Affiliations:** 1 School of Medicine, New York Medical College, 40 Sunshine Cottage Road, Valhalla, NY 10595 USA; 2 Department of Orthopaedic Surgery, Jefferson Health NJ, Cherry Hill, NJ USA; 3 New York Institute of Technology College of Osteopathic Medicine, Old Westbury, NY USA; 4 Department of Orthopaedic Surgery, Westchester Medical Center, Valhalla, NY USA; 5 Department of Orthopedic Trauma and Reconstructive Surgery, Grant Medical Center, Ohio Health, Columbus, OH USA

**Keywords:** Frailty, Intertrochanteric fracture, 5-Item modified frailty index, Fixation

## Abstract

**Purpose:**

This study sought to examine the role of frailty in predicting inpatient mortality following early surgical fixation of intertrochanteric (IT) fractures.

**Methods:**

The ACS-TQIP (2017–21) database was queried for patients with intertrochanteric fractures undergoing surgical fixation within 48 h. Frailty status was determined using the 5-item modified frailty index (mFI-5) (0 = nonfrail, 1 = prefrail, and, 2 = frail, and > 2 = severely frail). The primary end point was inpatient mortality. Secondary end points were unplanned ICU admission and thromboembolic events (DVT or PE). Significance was considered a *p*-value < 0.05.

**Results:**

Frailty, as determined by the mFI-5, was a significant predictor of inpatient mortality and unplanned ICU admissions. Frail and severely frail patients had higher odds of mortality (OR: 2.00 and 3.58, respectively) and ICU admission (OR: 1.66, 3.09, 3.83, respectively). When compared with nonoperative management in frail (OR: 0.561) and severely frail (OR: 0.665) patients, surgical intervention within 48 h significantly reduced the risk of mortality.

**Conclusion:**

Increasing frailty status, as measured by the mFI-5, has increased odds for unplanned ICU admission and inpatient mortality following surgery within 48 h for IT fractures. Although operative management is required in these patients, these results suggest frailty may be used to preoperatively identify patients at high risk for adverse events. Future studies may seek to identify modifiable factors in the preoperative period to improve outcomes in frail patients.

**Supplementary Information:**

The online version contains supplementary material available at 10.1007/s43465-026-01751-z.

## Introduction

Hip fractures in elderly patients often mark the beginning of an inevitable decline in functional capacity, subsequently increasing risk of mortality during both acute hospitalization and the post-discharge period. Previous studies have demonstrated 1-year mortality rates following a nonoperatively treated hip fracture as high as 83%, and even when surgically treated, 1-year mortality rate was about 25% [1–3]. Several factors have been implicated in the risk of postoperative mortality in this population, including ASA grade, nutritional status, frailty, and cardiac comorbidities [4–6]. Surgical fixation of hip fractures is preferred in these patients, as nonoperative management has been shown to dramatically increase rates of 1-year morbidity and mortality [3, 5, 7]. Specifically, operative intervention within 48 h has been associated with a decreased risk of mortality and adverse postoperative outcomes [5, 8].

Intertrochanteric (IT) fractures are more common in elderly patients who already have a lower baseline physiologic reserve and are prone to surgical complications [9]. One way to stratify this patient population is based on their frailty status, or the degree of decrease in functional reserve capacity and vulnerability to physiologic insults, including surgery. Frailty indices have previously been investigated as predictive metrics for adverse outcomes following orthopedic surgery [10–13]. Given that early surgical intervention is preferred as well as the utility of preoperative risk analysis, frailty may be a useful tool for this subset of patients. There is yet to be an investigation of frailty as a predictor of inpatient complications among patients receiving early surgical intervention for hip fractures. Utilizing the American College of Surgeons Trauma Quality Improvement Program (ACS-TQIP) database, we sought to elucidate the role of frailty as measured by the 5-item modified frailty index (mFI-5), in predicting inpatient mortality following early surgical fixation of IT fractures.

## Methods

### Data Collection

The ACS-TQIP database is a recognized registry designed to enhance the quality of trauma care through the utilization of a risk-adjusted benchmarking methodology. More than 875 hospitals participate in data gathering, and this database involves trained personnel abstracting patient and institutional variables. As ACS-TQIP contains de-identified patient information, this study did not require institutional review board approval. 2017–2021 data was collected using International Classification of Diseases, 10th revision, Clinical Modification (ICD-10-CM) diagnosis codes. Patients with fractures of the intertrochanteric region were included using ICD-10 diagnosis codes. Pathological fractures due to malignancy were excluded. Procedure codes for intramedullary fixation or open reduction and internal fixation were used to specify fixation type. All codes were collected using the 2023 HCUP ICD code directory. Patients presenting to affiliated trauma centers with fractures of the intertrochanteric region (S7214..) that were subsequently fixed surgically (OQS6..) were included. Fractures that were treated nonoperatively, fixed after 48 h, and cases with missing time to procedure data were excluded.

### mFI-5 frailty score

Frailty was assessed by identifying the variables used in the mFI-5, which include the following: congestive heart failure, chronic obstructive pulmonary disease (COPD), hypertension, diabetes, and non-independent functional status (requiring an assistive device for ambulation). The relevant variables are provided in the ACS-TQIP dataset, and patients were assigned one point for each diagnosis, and the total number of points for each patient was calculated. Patients were grouped into three categories based on their frailty score: 0 = nonfrail, 1 = prefrail, 2 = frail, and > 2 = severely frail [14].

### Covariates and Outcomes

The primary end point evaluated was inpatient mortality. Secondary end points were unplanned admission to the intensive care unit (ICU) and thromboembolic events such as pulmonary embolism (PE) or deep venous thrombosis (DVT). Additionally, data on race, sex, total charges, and primary payer were collected. Those older than 65 years were considered as elderly patients. Injury severity scores (ISS) were collected for each patient, as well as time to surgical fixation. Early surgery was considered within 48 h of trauma center arrival. A post hoc case–control analysis was conducted to compare frail and severely frail patients undergoing early surgical fixation with nonoperatively treated patients. One-to-one matching without replacement was performed based on age, sex, and ISS, selected a priori. Matching was conducted using exact matching for sex and nearest-neighbor matching for age and ISS. Balance between matched cohorts was assessed using Student’s *t*-tests and Chi-square tests (Supplemental 1–2).

### Statistical Analysis

Patients were grouped according to frailty status and demographic characteristics were assessed using a Chi-square test for categorical variables and a Kruskal–Wallis test for continuous variables. Categorical variables were presented as counts with percentages, and continuous variables were presented as medians with an interquartile range (IQR). Initially, a univariate regression was conducted for each variable selected a priori based on clinical relevance. Variables that were significantly associated with outcomes (*p* < 0.05) were used in a binary logistic regression and included age, sex, ISS, and ORIF versus IMN. Backward stepwise *p*-value removal was used to build the final model, and model fit was assessed using the area under the curve (AUC). Only preoperative variables were considered, and variables that were significant at or less than 0.05 were retained in the final model. We used the STROBE case–control checklist when writing our report [15]. All statistical analysis was performed using the Statistical Package for Social Sciences (SPSS, Version 29; IBM, Inc., Armonk, NY).

## Results

### Patient Demographics

There were a total of 41,213 patients who underwent surgical fixation of intertrochanteric fractures within 48 h of trauma center arrival. The median age was 76 years (IQR: 65–83), with 22,382 (54.3%) of the patients being female. The most common comorbidity was hypertension, with 25,049 (60.8%) patients affected. Additionally, there were 10,759 (26.1%) patients who required an assistive device for ambulation. In regard to frailty status as rated by the mFI-5, 10,099 (24.5%) patients were nonfrail, 14,667 (35.5%) were prefrail, 11,356 (27.6%) were frail, and 5091 (12.4%) were severely frail. The most common postoperative complications were unplanned admission to the ICU and inpatient mortality, with 799 (1.9%) and 587 (1.4%) patients affected, respectively. Postoperative complications included 230 (0.6%) patients with acute kidney injury, 145 (0.4%) with DVT, and 111 (0.3%) with PE. The median length of hospital stay was 5 days (IQR: 4–7 days) (Table 1).

**Table 1:** Baseline patient demographics and characteristics. Categorical variables are presented as counts with percentages and continuous variables are presented as medians with interquartile ranges

	Nonfrail (*n* = 10,099)	Prefrail (*n* = 14,667)	Frail (*n* = 11,356)	Severely frail (*n* = 5,091)
Age (median)	67.00 (IQR 26)	78.00 (IQR 16)	78.00 (IQR 14)	79.00 (IQR 13)
Female	5,252 (52.1%)	323 (56.8%)	6,122 (54.0%)	2,685 (52.8%)
*Race*				
White	8,858 (88.5%)	13,428 (92.0%)	11,309 (90.7%)	4,622 (91.0%)
Black	514 (5.1%)	508 (3.5%)	468 (4.1%)	212 (4.2%)
Hispanic	3,226 (33.3%)	4,982 (35.4%)	4,229 (38.5%)	2,122 (42.9%)
*Primary source of payment*				
Medicaid	608 (6.1%)	403 (2.8%)	277 (2.5%)	101 (2.0%)
Medicare	3,614 (36.2)	7,453 (51.2%)	5,698 (50.5)	2,452 (48.5%)
Private	1,903 (19.1%)	1,409 (9.7%)	864 (7.7%)	249 (4.9%)
*Complications*				
Mortality	71 (0.7%)	154 (1.0%)	199 (1.8%)	163 (3.2%)
DVT	39 (0.4%)	48 (0.3%)	34 (0.3%)	24 (0.5%)
AKI	20 (0.2%)	60 (0.4%)	88 (0.8%)	62 (1.2%)
ARDS	10 (0.1%)	14 (0.1%)	10 (0.09%)	6 (0.1%)
Cardiac arrest with CPR	23 (0.2%)	34 (0.2%)	38 (0.3%)	46 (0.9%)
MI	14 (0.1%)	35 (0.2%)	71 (0.6%)	43 (0.8%)
Superficial SSI	2 (0.02%)	2 (0.01%)	2 (0.02%)	2 (0.04%)
Deep SSI	7 (0.07%)	4 (0.03%)	2 (0.02%)	1 (0.02%)
Extremity compartment syndrome	6 (0.06%)	0 (0%)	0 (0%)	0 (0%)
Organ/space SSI	1 (0.01%)	0 (0%)	0 (0%)	0 (0%)
PE	40 (0.4%)	27 (0.2%)	23 (0.2%)	21 (0.4%)
Stroke/CVA	25 (0.2%)	66 (0.4%)	52 (0.5%)	27 (0.5%)
Unplanned intubation	28 (0.3%)	55 (0.4%)	72 (0.6%)	53 (1.0%)
Osteomyelitis	1 (0.01%)	0 (0%)	1 (0.009)%	0 (0%)
Unplanned ICU admission	102 (1.0%)	209 (1.4%)	308 (2.7%)	180 (3.5%)
Unplanned return to OR	26 (0.2%)	24 (0.2%)	17 (0.1%)	11 (0.2%)
Severe sepsis	6 (0.06%)	22 (0.1%)	32 (0.3%)	14 (0.3%)
Catheter-associated UTI	8 (0.08%)	23 (0.2%)	16 (0.1%)	6 (0.1%)
Central line-associated bloodstream infection	4 (0.03%)	1 (0.007%)	1 (0.009%)	1 (0.02%)
Ventilator-associated pneumonia	6 (0.06%)	4 (0.03%)	5 (0.04%)	2 (0.04%)
Pressure ulcer	19 (0.2%)	35 (0.2%)	34 (0.3%)	29 (0.6%)
comorbidities				
Current smoker	2,164 (21.4%)	1,972 (13.4%)	1,519 (13.4%)	802 (15.8%)
Diabetes mellitus	0 (0%)	1,130 (7.7%)	4,338 (38.2%)	3,218 (63.2%)
Hypertension	0 (0%)	9,990 (68.1%)	10,198 (89.8%)	4,861 (95.5%)
CHF	0 (0%)	233 (1.6%)	1,018 (9.0%)	2,126 (41.8%)
COPD	0 (0%)	1,108 (7.6%)	2,306 (20.3%)	2,463 (48.4%)
Functionally Dependent health status	0 (0%)	2,206 (15.0%)	4852 (42.7%)	3701 (72.7%)
IMN	7,315 (72.4%)	11,276 (76.9%)	8,765 (77.2%)	4,060 (79.7%)
ORIF	2,802 (27.7%)	3,405 (23.2%)	2,593 (22.8%)	1,036 (20.3%)
Age > 65	4,931 (48.8%)	9,610 (65.5%)	7,567 (66.6%)	3,631 (71.3%)
Age > 75	3,074 (30.4%)	6,927 (47.2%)	5,499 (48.4%)	2,660 (52.2%)
ISS (median)	9.00 (IQR 1)	9.00 (IQR 0)	9.00 (IQR 0)	9.00 (IQR 1)
Days from arrival to discharge (median)	5.00 (3)	5.00 (3)	5.00 (3)	5.00 (4)
BMI (median)	23.88 (6.42)	24.31 (6.98)	25.11 (7.68)	25.71 (8.41)

### Multivariate Regression

A multivariate regression analysis was performed to understand the value of frailty as a predictor of unplanned ICU admission, inpatient death, and thromboembolic events. Frail and severely frail patients were at a significantly increased risk of mortality (frail = OR: 1.997, 95% CI 1.422–2.805, *p* < 0.001; severely frail = OR: 3.578, 95% CI 2.520–5.082, *p* < 0.001) when compared to nonfrail patients. Prefrail, frail, and severely frail patients were at significantly increased odds of unplanned admission to the ICU (prefrail = OR: 1.658, 95% CI 1.247–2.204, *p* < 0.001; frail = OR: 3.087, 95% CI 2.343–4.067, *p* < 0.001; severely frail = OR: 3.828, 95% CI 2.841–5.158, *p* < 0.001) compared to nonfrail patients. Increasing frailty was not a significant predictor of thromboembolic events including DVT and PE (Prefrail = OR: 0.983, 95% CI 0.603–1.602, *p* = 0.946; Frail = OR: 1.132, 95% CI 0.676–1.898, *p* = 0.637; Severely Frail = OR: 1.615, 95% CI 0.903–2.890, *p* = 0.106) (Tables 2, 3, 4).

**Table 2:** Multivariate analysis for inpatient death

Covariate	Odds ratio (95 CI)	*p*-Value
Age	1.051 (1.040–1.063)	< 0.001
Sex	0.908 (0.779–1.058)	0.217
ISS	1.100 (1.079–1.121)	< 0.001
Length of stay	1.043 (1.032–1.055)	< 0.001
Prefrail	1.092 (0.765–1.558)	0.629
Frail	1.997 (1.422–2.805)	< 0.001
Severely frail	3.578 (2.520–5.082)	< 0.001
ORIF versus IMN	0.976 (0.764–1.246)	0.845

Odds ratios presented with 95% confidence intervals. Significance was considered *p* < 0.05

**Table 3:** Multivariate analysis for unplanned ICU admission

Covariate	Odds ratio (95 CI)	*p*-Value
Age	1.012 (1.005–1.019)	< 0.001
Sex	0.857 (0.760–0.965)	0.011
ISS	1.021 (1.001–1.041)	0.041
Length of stay	1.106 (1.095–1.116)	< 0.001
Prefrail	1.658 (1.247–2.204)	< 0.001
Frail	3.087 (2.343–4.067)	< 0.001
Severely frail	3.828 (2.841–5.158)	< 0.001
ORIF versus IMN	0.864 (0.711–1.051)	0.144

Odds ratios presented with 95% confidence intervals. Significance was considered *p* < 0.05

**Table 4:** Multivariate analysis for DVT/PE

Covariate	Odds ratio (95 CI)	*p*-Value
Age	1.000 (0.988–1.012)	0.990
Sex	0.848 (0.653–1.101)	0.216
ISS	1.087 (1.062–1.112)	< 0.001
Length of stay	1.054 (1.042–1.066)	< 0.001
Prefrail	0.983 (0.603–1.602)	0.946
Frail	1.132 (0.676–1.898)	0.637
Severely frail	1.615 (0.903–2.890)	0.106
ORIF versus IMN	1.373 (0.948–1.988)	0.093

Odds ratios presented with 95% confidence intervals. Significance was considered *p* < 0.05

Case–control matching of frail and severely frail patients with nonoperatively treated counterparts demonstrated that operative management was associated with a significantly decreased risk of mortality in frail patients (OR: 0.561, 95% CI 0.457–0.688, *p* < 0.001) (Table 5). Operative management was associated with a significantly decreased risk of mortality (OR: 0.570, 95% CI 0.452–0.718, *p* < 0.001) and unplanned ICU admission (OR: 0.665, 95% CI 0.541–0.818, < 0.001) in severely frail patients.

**Table 5:** Multivariate for inpatient death among frail patients comparing nonoperative versus operative management

Covariate	Inpatient mortality		Unplanned ICU	
	OR (95% CI)	*p*-Value	OR (95% CI)	*p*-Value
Age	1.037 (1.025–1.049)	< 0.001	1.002 (0.994–1.011)	0.563
Female	0.588 (0.481–0.720)	< 0.001	0.803 (0.674–0.956)	0.014
ISS	1.117 (1.091–1.144)	< 0.001	1.073 (1.047–1.100)	< 0.001
Surgical fixation	0.561 (0.457–0.688)	< 0.001	0.890 (0.753–1.052)	0.173

Odds ratios presented with 95% confidence intervals. Significance was considered *p* < 0.05

## Discussion

Operative stabilization within 48 h of hospital admission is regarded as the standard of care for treatment of intertrochanteric hip fractures [5, 8]. As surgeons aim to intervene within this time frame, it is important to identify patients undergoing early fixation who are at risk of complications. In this study, we analyzed the TQIP database to explore the association between frailty as measured by the mFI-5 and postoperative complications following early surgical fixation of intertrochanteric hip fractures. The accuracy of predictiveness for mFI-5 regarding inpatient mortality following hip fracture fixation was also explored in this article. The findings in our study were threefold: (1) frail and severely frail patients are at a significantly increased risk of postoperative mortality and unplanned ICU admission following hip fracture fixation; (2) frailty is superior to age greater than 65 years for predicting immediate postoperative complications; (3) operative management is superior to nonoperative management among frail patients as rated by the mFI-5.

Numerous studies have explored the impact of time to surgical fixation on mortality. The benefits of operative management of hip fractures within 48 h are well established, however, risk stratification is still an important consideration for mitigation of adverse outcomes. The mFI-5 frailty index has been extensively validated to predict postoperative complications following orthopedic surgery [10–13]. Traven et al. showed that the mFI-5 is an independent predictor of postoperative morbidity and mortality following surgical fixation of hip fractures [16]. Similarly, Kim et al. demonstrated that frailty was an independent risk factor for adverse outcomes following hemiarthroplasty for femoral neck fractures [10]. Our study found that frail and severely frail patients (as rated by the mFI-5) undergoing early operative fixation were at an increased risk of mortality and unplanned ICU admission compared with their nonfrail and prefrail counterparts. Despite the well-established link between frailty and complications following hip fractures, frail patients undoubtedly represent a vulnerable subgroup.

Additionally, to determine the benefit of early surgery among frail patients in our study, we conducted a post hoc comparison of frail and severely frail patients receiving early surgery matched with a nonoperatively treated cohort. This demonstrates that the odds of mortality are significantly decreased in the surgical group. These findings are in line with several studies showing the superiority of operative management of hip fractures, including a study by Loggers et al., who found that nonoperative treatment of proximal femur fractures in frail patients was associated with an elevated risk of mortality [3]. Nonetheless, with increasing frailty comes an elevated risk of complications and morbidity. When a grave physiologic insult such as a hip fracture occurs in an already frail patient, the risk of complications is increased and typically unrelated to surgery [2, 3, 17, 18]. Despite this baseline elevated risk, operative management is still superior in this vulnerable patient population. With our findings in mind, calculation of an individualized frailty score can help identify patients with the highest likelihood of death if treated nonoperatively and who will thus significantly benefit from surgery. This tool can also be helpful with pre- and postoperative planning for those patients with an elevated risk of ICU admission.

Although this study sought to provide a comprehensive analysis of the early fixation of hip fractures in frail patients, it is not without limitations. This study's use of the TQIP database may limit the generalizability of the findings, as it only includes data from patients treated at registered trauma centers. Given the well-reported long-term risk of mortality associated with intertrochanteric hip fractures, we were unable to assess the impact of frailty beyond inpatient mortality in this study given the limitations of the data in TQIP. Moreover, residual confounders such as preoperative nutritional status, cognitive impairment, sarcopenia, end-of-life care decisions, and rehabilitation access are not considered in the mFI-5 and are unavailable with this database, but would be important to assess within the context of our results and mortality findings. Additionally, among the mFI-5 frailty categories, severely frail patients can have an mFI-5 score of 3, 4, or 5, increasing the variability of frailty status in this group. Further, as our analysis inherently relies on the validity of data entry, the accuracy of our results may be confounded by unknown errors, coding bias, and observer bias. There is also a risk of selection bias in the post hoc analysis, comparing frail surgical patients to nonoperative frail patients, as patients selected for surgery may have different baseline comorbidities and fracture severity that are not fully captured by age, sex, and ISS. Due to the variables available through the ACS-TQIP database, mFI-5 was used as a measure of frailty. Future studies should explore other available measures of frailty in this patient population, such as the Risk Analysis Index [19]. In terms of outcomes, unplanned ICU admission may not reflect the severity of complications consistently across trauma centers, as there is heterogeneity in regard to hospital policies, resource availability, and care models, such as closed versus open units. Results of thromboembolic events should also be interpreted with caution, as the incidence is low across frailty categories (0.3–0.5%). Despite these limitations, our study provides a novel use of mFI-5 in the stratification of frail patients based on early surgical fixation. To our knowledge, this analysis is the first to demonstrate that despite receiving early surgical fixation, a subgroup of this patient population is at unique risk of mortality. However, despite this risk, our study reports that surgical intervention is still associated with superior inpatient outcomes when compared to non-surgical management.

## Conclusion

Surgical fixation of intertrochanteric hip fractures within 48 h is widely accepted as the gold standard to reduce the increased risk of morbidity and mortality in this population. In this retrospective cohort study of a national trauma database, we demonstrate that increasing frailty as measured by the mFI-5 has increased odds for unplanned ICU admission and inpatient mortality. Although urgent surgical management is often necessary for IT fractures, the mFI-5 may serve as a useful tool for preoperative risk stratification and postoperative preparedness with at-risk patients. Future studies should seek to validate these results in a prospective cohort to understand the optimal management of intertrochanteric hip fractures in frail patients.

## Supplementary Information

Below is the link to the electronic supplementary material.

Supplementary file1 (DOCX 25 KB)

## Data Availability

Data can be made available upon request from the authors.
