# Supplementary material for: Frailty Is a Predictor of Inpatient Mortality and Unplanned ICU Admission Following Early Fixation of Intertrochanteric Fractures
Source: Indian J Orthop. 2026 Mar 12;60(5):1237–43. doi: 10.1007/s43465-026-01751-z (PMC13219681; doi:10.1007/s43465-026-01751-z)
Supplement: Supplementary file 1 — Supplementary file1 (DOCX 25 KB) [file 43465_2026_1751_MOESM1_ESM.docx]

**Supplementary Tables**

Supplemental 1. Baseline patient demographics for frail patients, operative and nonoperative treatment matched cohorts based on age, sex, and injury severity score.

| Variables | No Surgery (n = 9826) | Surgery (n = 9826) | p-value |
| --- | --- | --- | --- |
| Age (Median) | 78.00 | 78.00 | 1.000 |
| Female | 6465 (65.8%) | 6465 (65.8%) | 0.506 |
| Comorbidities |  |  |  |
| COPD | 2332 (23.7%) | 2269 (23.1%) | 0.148 |
| CHF | 1104 (11.2%) | 806 (8.2%) | **<0.001** |
| Hypertension | 8673 (88.3%) | 8806 (89.6%) | **0.001** |
| Functionally Dependent | 3260 (33.2%) | 3436 (35.0%) | **0.004** |
| Diabetes Mellitus | 4283 (43.6%) | 4335 (44.1%) | 0.232 |
| Complications |  |  |  |
| Death | 261 (2.7%) | 149 (1.5%) | **<0.001** |
| Unplanned Return to OR | 16 (0.2%) | 21 (0.2%) | 0.255 |
| AKI | 72 (0.7%) | 70 (0.7%) | 0.466 |
| ARDS | 21 (0.2%) | 9 (0.1%) | **0.022** |
| Cardiac Arrest with CPR | 91 (0.9%) | 33 (0.3%) | **<0.001** |
| Deep SSI | 2 (0.02%) | 3 (0.03%) | 0.500 |
| DVT | 44 (0.4%) | 38 (0.4%) | 0.290 |
| Extremity Compartment Syndrome | 3 (0.03%) | 0 (0%) | 0.125 |
| MI | 49 (0.5%) | 45 (0.5%) | 0.378 |
| Organ/Space SSI | 2 (0.02%) | 0 (0%) | 0.250 |
| PE | 34 (0.3%) | 24 (0.2%) | 0.118 |
| Stroke/CVA | 49 (0.5%) | 45 (0.5%) | 0.378 |
| Unplanned Intubation | 103 (1.0%) | 68 (0.7%) | **0.005** |
| Osteomyelitis | 1 (0.01%) | 1 (0.01%) | 0.750 |
| Unplanned ICU | 300 (3.1%) | 268 (2.7%) | 0.093 |
| Severe Sepsis | 46 (0.5%) | 27 (0.3%) | **0.017** |
| Catheter-Associated UTI | 18 (0.2%) | 9 (0.09%) | 0.062 |
| Central Line-Associated Bloodstream Infection | 1 (0.01%) | 2 (0.02%) | 0.500 |
| Ventilator-Associated Pneumonia | 11 (0.1%) | 7 (0.1%) | 0.240 |
| Alcohol Withdrawal Syndrome | 50 (0.5%) | 34 (0.3%) | 0.050 |
| Pressure Ulcer | 57 (0.6%) | 37 (0.4%) | **0.025** |
| Superficial SSI | 1 (0.01%) | 1 (0.01%) | 0.750 |
| Race |  |  |  |
| White | 8589 (88.0%) | 8778 (89.7%) |  |
| Black | 527 (5.4%) | 467 (4.8%) |  |
| Hispanic | 630 (6.7%) | 518 (5.5%) |  |
| Primary Source of Payment |  |  |  |
| Medicaid | 405/9743 (4.2%) | 302/9761 (3.1%) |  |
| Medicare | 6393/9743 (65.6%) | 4815/9761 (49.3%) |  |
| Private Insurance | 1079/9743 (11.1%) | 862/9761 (8.8%) |  |
| ISS (Median) | 9.00 | 9.00 | 1.000 |
| LOS (Median) | 6.00 | 5.00 | **<0.001** |

Supplemental 2. Baseline patient demographics for severely frail patients, operative and nonoperative treatment matched cohorts based on age, sex, and injury severity score.

| Variables | No Surgery (n = 4513) | Surgery (n = 4513) | p-value |
| --- | --- | --- | --- |
| Age (Median) | 79.00 | 79.00 | 1.000 |
| Female | 2879 (63.8%) | 2879 (63.8%) | 0.509 |
| Comorbidities |  |  |  |
| COPD | 2260 (50.1%) | 2315 (51.3%) | 0.128 |
| CHF | 1918 (42.5%) | 1754 (38.9%) | **<0.001** |
| Hypertension | 4291 (95.1%) | 4306 (95.4%) | 0.244 |
| Functionally Dependent | 3150 (69.8%) | 3142 (69.6%) | 0.436 |
| Diabetes Mellitus | 3025 (67.0%) | 3026 (67.1%) | 0.500 |
| Complications |  |  |  |
| Death | 202 (4.5%) | 118 (2.6%) | **<0.001** |
| Unplanned Return to OR | 15 (0.3%) | 10 (0.2%) | 0.212 |
| AKI | 72 (1.6%) | 56 (1.2%) | 0.091 |
| ARDS | 4 (0.1%) | 7 (0.2%) | 0.274 |
| Cardiac Arrest with CPR | 62 (1.4%) | 40 (0.9%) | **0.018** |
| Deep SSI | 0 (0%) | 1 (0.02%) | 0.500 |
| DVT | 29 (0.6%) | 22 (0.5%) | 0.200 |
| MI | 39 (0.9%) | 37 (0.8%) | 0.454 |
| Organ/Space SSI | 1 (0.02%) | 0 (0%) | 0.500 |
| PE | 19 (0.4%) | 19 (0.4%) | 0.564 |
| Stroke/CVA | 32 (0.7%) | 24 (0.5%) | 0.174 |
| Unplanned Intubation | 91 (2.0%) | 50 (1.1%) | **<0.001** |
| Osteomyelitis | 2 (0.04%) | 0 (0%) | 0.250 |
| Unplanned ICU | 233 (5.2%) | 158 (3.5%) | **<0.001** |
| Severe Sepsis | 26 (0.6%) | 16 (0.4%) | 0.082 |
| Catheter-Associated UTI | 13 (0.3%) | 4 (0.1%) | **0.024** |
| Central Line-Associated Bloodstream Infection | 0 (0%) | 1 (0.02%) | 0.500 |
| Ventilator-Associated Pneumonia | 4 (0.1%) | 0 (0%) | 0.062 |
| Alcohol Withdrawal Syndrome | 13 (0.3%) | 14 (0.3%) | 0.500 |
| Pressure Ulcer | 47 (1.0%) | 24 (0.5%) | **0.004** |
| Superficial SSI | 2 (0.04%) | 1 (0.02%) | 0.500 |
| Race |  |  |  |
| White | 3915 (87.0%) | 4077 (90.6%) |  |
| Black | 294 (6.5%) | 204 (4.5%) |  |
| Hispanic | 287 (6.6%) | 230 (5.3%) |  |
| Primary Source of Payment |  |  |  |
| Medicaid | 104 (2.3%) | 117 (2.6%) |  |
| Medicare | 1743 (39.0%) | 2153 (48.0%) |  |
| Private Insurance | 221 (4.9%) | 237 (5.3%) |  |
| ISS (Median) | 9.00 | 9.00 | 0.600 |
| LOS (Median) | 7.00 | 6.00 | **<0.001** |
